# Associations Between Indoor Fungal Community Structures and Environmental Factors: Insights from the Evidence-Driven Indoor Air-Quality Improvement Study

**DOI:** 10.3390/jof11040261

**Published:** 2025-03-28

**Authors:** Iva Šunić, Dubravka Havaš Auguštin, Jelena Šarac, Kristina Michl, Tomislav Cernava, Rasmus Riemer Jakobsen, Armin Mešić, Natalija Novokmet, Mario Lovrić

**Affiliations:** 1 Centre for Applied Bioanthropology, Institute for Anthropological Research, 10000 Zagreb, Croatia; iva.sunic@inantro.hr (I.Š.); jsarac@inantro.hr (J.Š.); nnovokm@inantro.hr (N.N.); mario.lovric@inantro.hr (M.L.); 2 Faculty of Biotechnology and Drug Development, University of Rijeka, 51000 Rijeka, Croatia; 3 Institute of Environmental Biotechnology, Graz University of Technology, 8010 Graz, Austria; kristina.michl@tugraz.at (K.M.); tomislav.cernava@tugraz.at (T.C.); 4 School of Biological Sciences, Faculty of Environmental and Life Sciences, University of Southampton, Southampton SO17 1BJ, UK; 5 Department of Food Science, University of Copenhagen, 1958 Copenhagen, Denmark; rasmus@food.ku.dk; 6 Institute Ruđer Bošković, 10000 Zagreb, Croatia; Armin.Mesic@irb.hr; 7 The Lisbon Council, 1040 Brussels, Belgium

**Keywords:** bed dust microbiome, Illumina sequencing, fungal ITS, environmental factors, Evidence-Driven Indoor Air-Quality Improvement project

## Abstract

Indoor fungal communities, found in household dust, significantly influence indoor air quality and health. These communities are shaped by environmental, socioeconomic, and household factors. However, studies on indoor mycobiomes, particularly in Croatia, remain limited. This study investigates the relationship between environmental and household factors and indoor fungal communities, focusing on their diversity, composition, and potential health impacts in Croatian households. Dust samples from 66 Croatian households were analyzed using fungal ITS sequencing. Statistical analyses, including alpha diversity measures, were conducted to evaluate the influence of variables such as pet ownership, number of siblings, and cleaning habits on fungal diversity and abundance. Dominant genera included *Malassezia*, *Cladosporium*, and the family *Didymosphaeriaceae*. Pet ownership and sibling presence were linked to higher fungal diversity, with outdoor-associated genera such as *Aureobasidium* being more abundant in these households. Cleaning practices selectively altered fungal communities, with frequent cleaning reducing diversity, but not eliminating resilient taxa like *Malassezia*. This study highlights the interplay between environmental, household, and socioeconomic factors in shaping indoor fungal communities. The findings underscore the importance of addressing indoor fungal diversity to improve air quality and health, particularly in households with vulnerable populations.

## 1. Introduction

Household dust serves as a major reservoir of microbial communities, including bacteria, fungi, and viruses. As dust particles become resuspended into the air, they carry these microorganisms with them, potentially influencing indoor air quality and exposure. The presence of different microorganisms, such as fungi, has been linked to both protective and detrimental health effects, especially respiratory conditions such as asthma and allergies [1,2,3,4,5,6]. Most indoor fungal communities primarily originate from outdoor environments and are influenced by geographic, climatic, and vegetative factors [1,7,8]. Commonly identified genera in house dust include *Cladosporium*, *Aspergillus*, *Penicillium*, *Alternaria*, *Epicoccum*, *Phoma*, *Saccharomyces*, *Aureobasidium*, *Cryptococcus*, *Rhodotorula*, *Cyberlindnera*, and *Candida*, which together represent a wide range of fungal diversity. These genera are associated with both indoor and outdoor environments and exhibit considerable adaptability [1,2,9,10]. Rare and moisture-associated fungal genera such as *Malassezia* (associated with human skin), *Sphaerellopsis*, *Curvularia*, and *Aureobasidium*, as well as yeasts like *Cryptococcus* and *Saccharomyces*, further contribute to the taxonomic variety within indoor dust [11,12,13] together with molds such as *Alternaria alternata* and *Cladosporium cladosporioides*, which are also frequently linked to respiratory symptoms [14,15]. In total, the diversity of fungi in house dust encompasses a vast range of taxa. This diversity is, on average, approximately 50% higher indoors than outdoors, likely due to indoor environments harboring both outdoor-derived microbes and taxa specific to indoor sources [7].

The indoor microbial environment is influenced by multiple factors, including the inhabitants, their activities, and the interaction between indoor and outdoor spaces [14]. Fungal communities, in particular, are shaped by complex interactions between external environmental factors and household-specific characteristics, such as cleaning practices, pet ownership, and the number of occupants [16,17,18,19]. Higher microbial diversity is typically observed in rural or lower socioeconomic areas, while urbanization often reduces fungal diversity indoors [7,11,20]. Pet ownership, particularly of dogs, has been shown to increase fungal diversity indoors [18,20]. Larger households, especially those with multiple children, also exhibit greater microbial diversity [15,20]. Conversely, over-cleaning and reduced dust accumulation lower microbial diversity, with carpeted environments harboring more fungi than smooth flooring [21]. This variation in mycobiota composition is also reflected in fungal taxa associated with different household surfaces. For instance, *Eurotium repens* and *Penicillium chrysogenum* are frequently found in carpet dust, whereas *Cladosporium* and *Aspergillus* species are more prevalent in floor dust [14].

In this study, conducted as part of the EDIAQI project (Evidence-Driven Indoor Air Quality Improvement), we collected house dust samples from 66 households in the Zagreb region of Croatia and performed fungal amplicon sequencing, making it the first study of its kind in Croatia [22]. Our aim is to understand the relationship between environmental factors and the indoor mycobiome, as well as how the indoor environment shapes fungal communities in households. Given the increasing evidence linking indoor microbial exposure to respiratory issues, allergies, and other health effects [4,5,6,11,14,17,20,23,24,25], understanding these microbial communities is essential for assessing potential health risks. While this study focuses on the preliminary characterization of fungal communities, future research will incorporate respiratory health data to explore associations between indoor fungal exposure and respiratory conditions.

## 2. Materials and Methods

### 2.1. Study Group and Sample Collection

**Figure 1:**
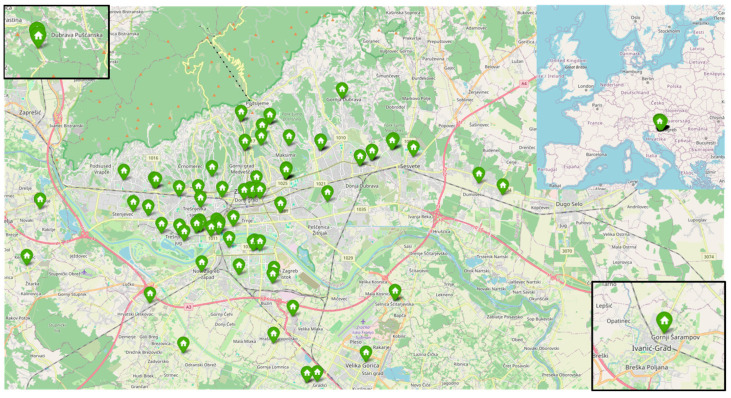
Geographic distribution of the households included in the Zagreb pilot study.

**Figure 2:**
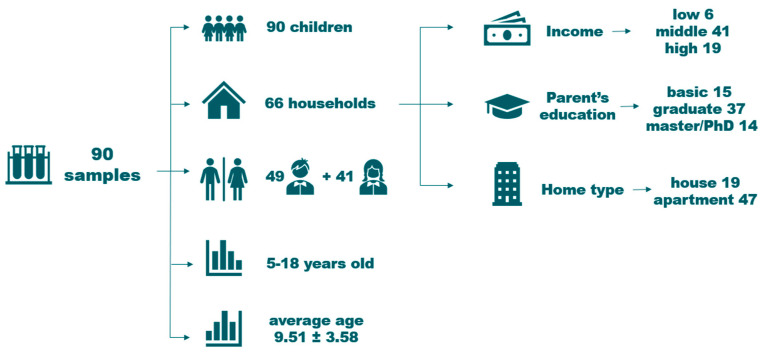
Households and participants overview.

**Figure 3:**
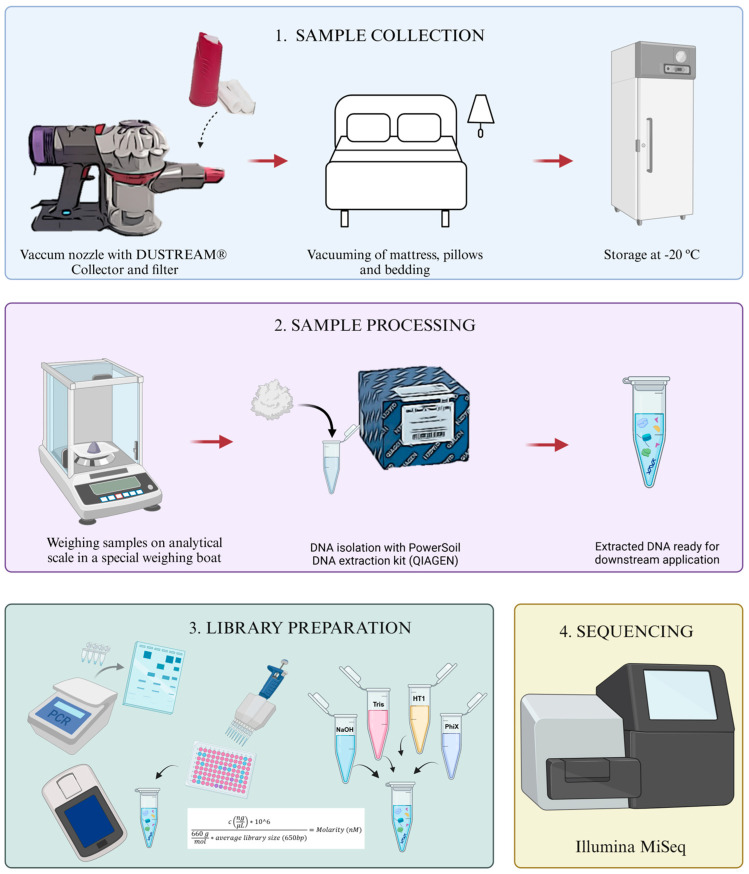
Illustration of the dust collection and isolation protocol in the Zagreb pilot.

The samples were taken at 66 households from the EDIAQI’s Zagreb pilot study (Figure 1). Participants were enrolled during routine medical examinations at Children’s Hospital Srebrnjak, through public calls in newspapers, social media, and volunteers who willingly enrolled their children in the project. The map illustrates the geographic distribution of these households across the Zagreb County. The samples were collected from children’s mattresses. Household characteristics and socioeconomic status are presented in Figure 2. The recruited group consists of 90 children, with a gender ratio of 49 boys and 41 girls. The age range spans from 5 to 18 years, with an average age of 9.51 (±3.81). Dust samples were collected from children’s bedding using DUSTREAM^®^ Collector vacuum cleaner filters (Indoor Biotechnologies, Cardiff, UK) that were UV-light-radiated for 30 min before usage and placed on the vacuum cleaner nozzle to collect bed dust. Samples were collected by trained laboratory personnel, and the time required for each bed was approximately 5 min, including vacuuming of mattresses, pillows, and bedding. The filters with samples were then stored at −20 °C until DNA isolation to preserve genetic material and eliminate dust mites. For the microbial DNA isolation, dust samples were weighed on an analytical scale Axis ALN120 (Axis, Gdańsk, Poland) and aliquoted to the target mass of each sample to be around 60–80 mg. This amount of dust was chosen after several trial isolations, and has been shown to have the highest DNA yield. The complete process and protocols are outlined in Figure 3.

### 2.2. Isolation of Fungal DNA

The genetic material was isolated using a DNeasy^®^ PowerSoil^®^ Pro Kit (Qiagen, Hilden, Germany), which can be used to extract microbial genomic DNA from all soil types and difficult samples such as sediment or dust [26]. Isolation steps were performed following the manufacturer’s protocol. The concentration of isolated genomic DNA was checked using a Qubit 3.0Fluorometer by Qubit dsDNA BR Assay Kit (Invitrogen-Thermo Fisher Scientific, Eugene, OR, USA), with concentrations ranging from 5.24 to 135 ng/µL. The isolated DNA was stored at −20 °C until further laboratory procedures.

### 2.3. Library Preparation and Sequencing

The library preparation was performed following the Illumina Metagenomic Sequencing Library Preparation protocol [27]. All DNA isolates were then diluted and normalized to 5 ng/µL. Diluted samples were amplified using a PCR Agilent SureCycler 8800 (Agilent Technologies, Santa Clara, CA, USA) with specific sets of primers for fungi. The internal transcribed spacer, region 2 (ITS2) was targeted using primers gITS7F (5′-TCG TCG GCA GCG TCA GAT GTG TAT AAG AGA CAG GTG ART CAT CGA RTC TTT G-3′) and ITS4ngs (5′-GTC TCG TGG GCT CGG AGA TGT GTA TAA GAG ACA GTT CCT SCG CTT ATT GAT ATG C-3′), as reported previously by Gupta et al., 2020 [11]. All PCR reactions were performed with a KAPA HiFi HotStart Ready Mix (Roche, Basel, Switzerland, CHE), including template-free PCR grade water as a negative control and ZymoBIOMICS Microbial Community DNA Standard (Zymo Research, Irvine, CA, USA) as a positive control. The PCR protocol was as follows: initial denaturation at 95 °C for 3 min followed by 30 cycles of 95 °C for 30 s, 55 °C for 30 s, and 72 °C for 30 s, and a final step of 72 °C for 5 min. After amplification, the quality of PCR products and the size of the fragments were verified on the automated electrophoresis platform 4200 TapeStation System (Agilent Technologies, Santa Clara, CA, USA). The next step included the clean-up of PCR products using MagSi-NGSPREP Plus* beads (Magtivio, Nuth, The Netherlands), based on magnetic bead technology and freshly prepared 80% ethanol. In the second step, primers with sequencing adaptors (Nextera XT Index Kit v2, Illumina, San Diego, CA, USA) were used for sample multiplexing so that each sample could be identified in the post-sequencing analysis. The same program as in the first PCR step was used, but with a reduced number of cycles (15 cycles). PCR products were also cleaned with magnetic beads and ethanol, and the libraries were again quantified using Qubit 3.0, and then normalized and pooled using the procedure described in the Illumina 16S Metagenomic Sequencing Library Preparation protocol. Pooled libraries were denatured with 0.2 N NaOH and adjusted to a final concentration of 8 pM. The run included 5.0% PhiX as internal control. Sequencing was performed using the MiSeq Reagent Kit v3 (600 cycles) on the Illumina MiSeq System (Illumina Inc., CA, USA).

### 2.4. Statistical Analysis

#### 2.4.1. Data Preprocessing and Taxonomic Classification

Data preprocessing and taxonomic classification were performed using the obtained ITS fastq files and were further processed using previously established bioinformatic pipelines [28]. Primers were removed from the raw sequences using Cutadapt [29]. Quality filtering, the removal of chimeric sequences, and the generation of amplicon sequence variants (ASVs) were performed using the DADA2 algorithm within the QIIME2 environment [30,31]. Quality control was performed using the q2-dada2 plugin within the QIIME2 environment, following its default parameters. The denoise_paired action was used to conduct quality filtering, chimera removal, and paired-end read joining. Following the DADA2 guidelines [30] for ITS data processing removal of reads for ASV length standardization and sequence trimming were not performed. The ASVs were then classified using the vsearch algorithm for sequence alignment and taxonomy assignment [32]. Fungal ITS sequences were classified by reference to the UNITE database [33], ensuring the accurate identification and classification of the microbial diversity and composition within the samples.

#### 2.4.2. Statistical Analysis

All analyses were conducted in R [version 4.3.0], primarily using the phyloseq [34], ggplot2 [35], rabuplot [36], and microeco [37] packages. Data visualization was customized to ensure clarity and reproducibility. Initial data pre-processing and filtering were performed using the phyloseq package in R. Taxonomic reads unassigned at the phylum level were identified and excluded to ensure only high-confidence fungal sequences were retained. This step removed approximately 15% of the total reads, with manual BLAST (version 2.14.1) searches indicating that most were human genomic DNA contaminants. Next, rare and low-abundance amplicon sequence variants (ASVs) were filtered out to reduce noise and focus on biologically meaningful taxa. ASVs present in fewer than three samples or with fewer than 1000 reads in total were excluded. This prevalence and abundance thresholding retained 88% of the original reads, representing most of the microbial diversity while ensuring statistical robustness.

After collapsing the dataset to summarize ASVs under their corresponding genera, taxonomic abundance was then analyzed at the genus level. Bar plots were generated to visualize the relative abundance of taxa across samples and grouped by key variables, such as the number of siblings and parental education level. A sample with unusually low observed ASV count was identified and removed to avoid skewing the results. Genus-level bar plots revealed the dominant taxa in the dataset. The statistical tests used by the function depend on the structure of the data and the number of categories in the predictor. In the case of two groups (pet ownership variable: “Yes” vs. “No”), a non-parametric Wilcoxon rank-sum test was used, while comparisons involving more than two groups (number of siblings variable: “0”, “1” and “2 or more”) used Kruskal–Wallis tests. To ensure the reliability of results when testing multiple microbial taxa simultaneously, the analysis applied a multiple testing correction to control the false discovery rate. In the end, these statistical methods were integrated into the visualization workflow, which shows comprehensive and precise assessment of the effects of categorical variables on microbial community composition.

Alpha diversity was assessed using species ASV richness. Statistical comparisons were performed using the same approach as described above, with the Wilcoxon rank-sum test for binary categorical variables and the Kruskal–Wallis test for variables with three or more categories. To correct for multiple testing, False Discovery Rate (FDR) adjustment was applied to the *p*-values.

#### 2.4.3. Covariates

Parental income, built environment, number of siblings, cleaning practices, and pet ownership were assessed using questionnaires completed by the parents. The questionnaires were designed by trained psychologists from the Institute for Anthropological Research and validated on a representative sample. The educational level was classified into three categories: Basic (Secondary Vocational Education and Higher Vocational Education or Undergraduate Degree), Graduate (High Vocational Education, including completed graduate studies, master’s degree, or professional postgraduate specialist study), and Master/PhD (Master of Science or Doctor of Science). Income was defined as the total monthly household income. The living environment was categorized as Suburban, Urban built aera (city, within a built environment), and Urban green area (city, surrounded by green spaces), while housing type was classified as either house or apartment. Dusting furniture was categorized into three frequencies: occasionally, once a week, and frequently (four or more times per week). Mattress vacuuming was categorized as never or once a year, occasional (from two to four times a year), and frequent (more than four times a year). The sampling was conducted only once per child’s mattress, with the sample collection periods divided into the heating season (October–March) and the non-heating season (April–September). Table 1 details the distribution and number of participants across these categories.

## 3. Results

### 3.1. Taxonomic Composition

Samples were analyzed by ITS rRNA gene sequencing, and all samples (90) were included after quality control. First, we examined the overall composition of samples and the most prominent taxa in terms of relative abundance. The most prominent genera were *Malassezia*, Unassigned *Didymosphaeriaceae*, and *Cyberlindnera*, with less abundant genera grouped under “Other”. *Malassezia* is the dominant genus in many samples, followed by Unassigned *Didymosphaeriaceae* (family *Didymosphaeriaceae*) and *Cyberlindnera*. The category ‘Unassigned Unassigned’ represents sequences that could not be classified at any taxonomic level, including genus, family, or higher levels, based on the reference database used. These sequences likely correspond to taxa that are either not represented or poorly represented in the reference database or may result from low-confidence taxonomic assignments. In contrast, categories like ‘Unassigned *Didymosphaeriaceae*’ indicate sequences that were assignable to the family *Didymosphaeriaceae*, but not to a specific genus within that family. Additional genera, such as *Aspergillus*, *Alternaria*, and *Cladosporium*, appear in smaller proportions. These distribution patterns indicate that, while *Malassezia* dominates, other genera create a more diverse, although less abundant, microbial profile. This variability in fungal composition may be influenced by various environmental or individual factors affecting microbial abundance within the studied group.

### 3.2. Alpha Diversity of Fungal Communities in Relation to Environmental and Socioeconomic Factors

**Figure 4:**
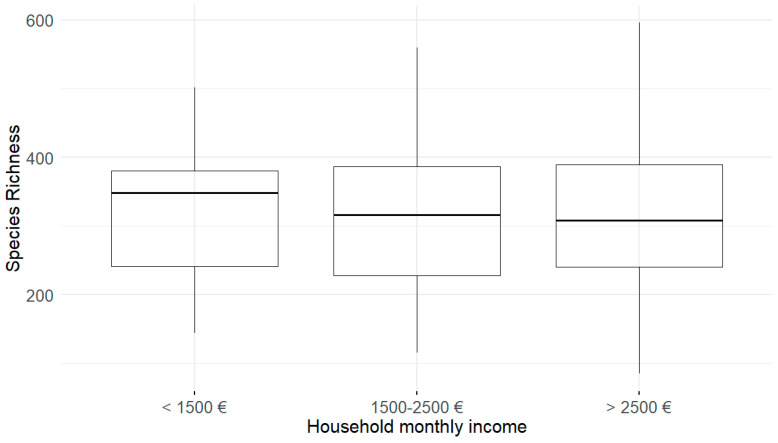
Alpha diversity (species richness) by household income.

Alpha diversity, measured by species richness, was compared across demographic, socioeconomic, and environmental factors (Table 1). Household monthly income was the only factor significantly associated with alpha diversity (Kruskal–Wallis chi-squared (χ^2^) = 6.451, df = 2, *p* = 0.018, Figure 4). The lowest income group (EUR < 1500) had the highest median richness and a relatively wide variability. The middle-income group (EUR 1500–2500) had a slightly lower median richness and the highest variability, though both very similar to the highest income group (EUR > 2500). Other factors, including parental education, living environment, home type, number of siblings, pet ownership, dusting frequency, mattress vacuuming, and sample collection season, showed no significant associations (all *p*-values > 0.05).

### 3.3. Influence of Pet Ownership and Sibling Count

**Figure 5:**
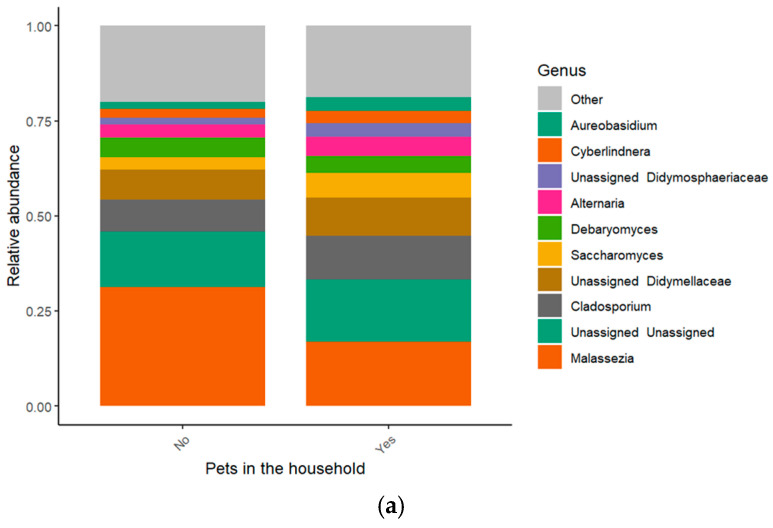

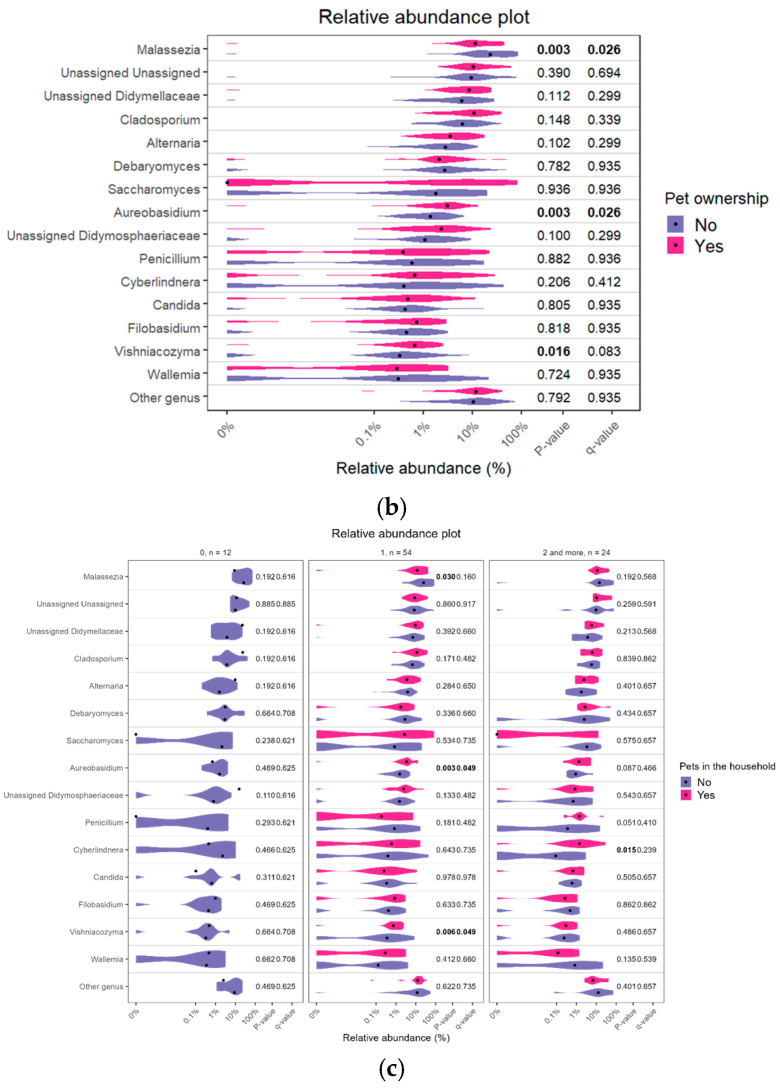
Relative abundance of microbial genera by pet ownership. (**a**) Stacked bar plot highlights the top ten genera, with others grouped as “Other”, ordered by mean abundance across samples. (**b**) Violin plot illustrates genus-level distribution and variability, with *p*-values indicating significant differences between groups. (**c**) Associations of siblings and pet ownership with fungal genera. Influence of number of siblings and pet ownership on the relative abundance of fungal genera.

Pet ownership influenced fungal community composition. Households with pets showed a higher abundance of *Aureobasidium* (Wilcoxon rank-sum test, W = 544, *p* = 0.003, r = −0.011, CI 95% −0.020 to −0.003) and *Vishniacozyma* (W = 605, *p* = 0.016, r = −0.002, ICI 95% −0.004 to −0.0004), while *Malassezia* was more dominant in homes without pets, (W = 1229.5, *p* = 0.003, r = 0.082, CI 95% 0.026 to 0.178; Figure 5a,b). The interaction between sibling count and pet ownership (Figure 5c) also shaped fungal composition. Homes with one sibling showed significant changes in abundances of *Malassezia* (Wilcoxon rank-sum test, W = 431, *p* = 0.03, r = 0.099, CI 95% 0.012 to 0.244), *Aureobasidium* (W = 156, *p* = 0.003, r = −0.015, CI 95% −0.027 to −0.005), and *Vishniacozyma* (W = 168, *p* = 0.006, r = −0.004, CI 95% −0.008 to −0.001). Households with two or more siblings and pets displayed marginally significant increased abundances of *Penicillium* (W = 38, *p* = 0.051, r = −0.008, CI 95% −0.014 to 0.0007) and *Aureobasidium* (W = 42, *p* = 0.087, r = −0.006, CI 95% −0.028 to 0.001), while those with no siblings and no pets exhibited lower overall fungal diversity. Cyberlindnera (W = 30, *p* = 0.0015, r = −0.012, CI 95% −0.033 to −0.001) was notably abundant in households with two or more siblings and pets.

### 3.4. Impact of Cleaning Practices

**Figure 6:**
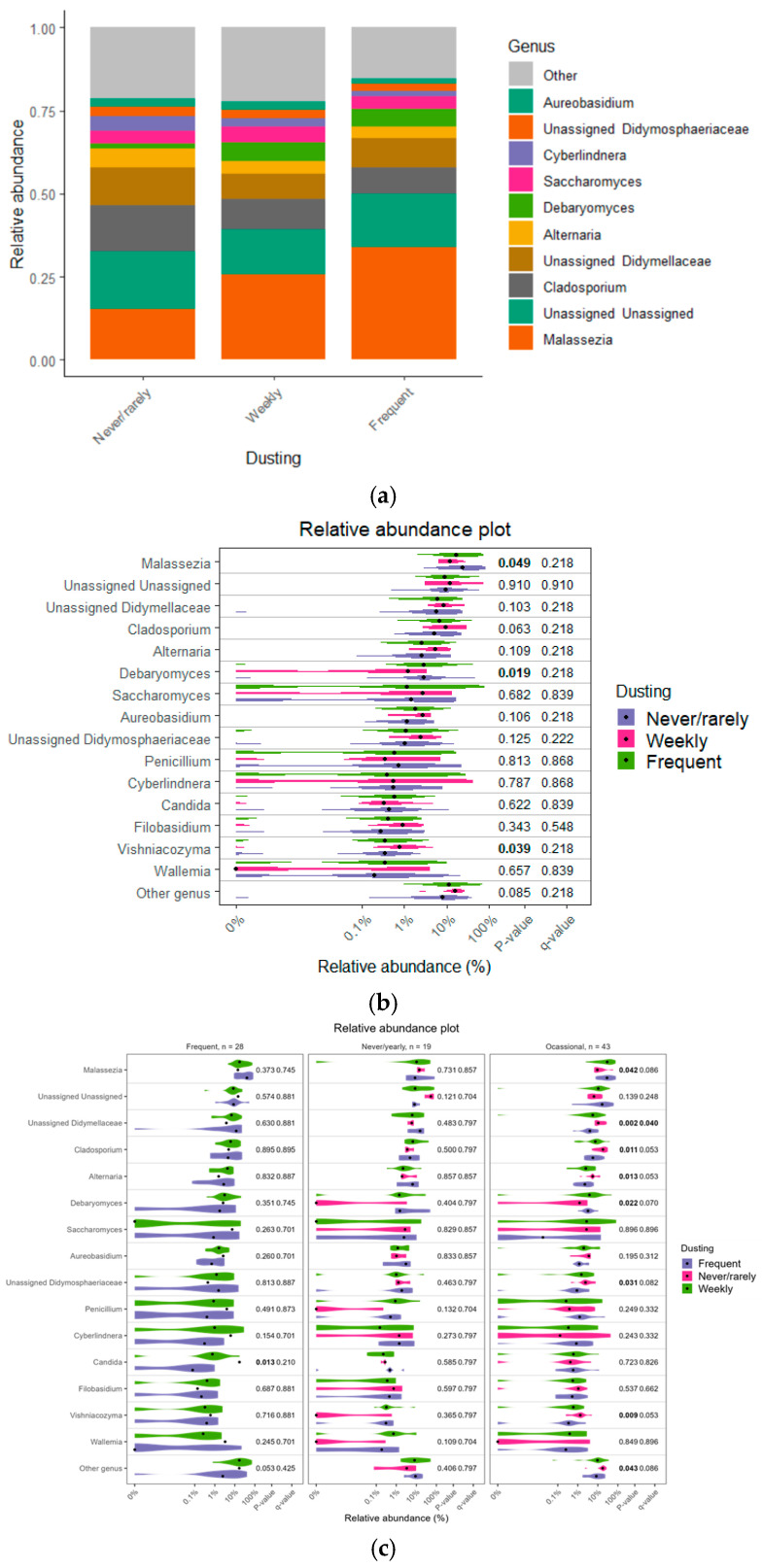
Relative abundance of microbial genera by dusting frequency. (**a**) Stacked bar plot highlights the top ten genera, with others grouped as “Other”, ordered by mean abundance across samples. (**b**) Violin plot illustrates genus-level distribution and variability, with *p*-values indicating significant differences between groups. (**c**) Interaction between vacuuming mattress frequency and dusting in the household on the relative abundance of fungal genera.

Infrequent dusting was associated with reduced abundances of *Malassezia* (Kruskal–Wallis chi-squared (χ^2^) = 6.048, df = 2, *p* = 0.049) and *Debaryomyces* (χ^2^ = 7.924, df = 2, *p* = 0.019). While *Cyberlindnera*, *Aureobasidium*, family *Didymellaceae*, and other less common genera categorized as “Other genera” also showed reduced trends in relative abundance, visually, these differences were not statistically significant (Figure 6a,b). In contrast, *Vishniacozyma* (χ^2^ = 6.513, df = 2, *p* = 0.039) showed a slightly higher abundance in households with frequent dusting. The interactions between cleaning practices (Figure 6c) show that infrequent cleaning practices favor many taxa: *Malassezia* (χ^2^ = 3.256, df = 2, *p* = 0.043), family *Didymellaceae* (χ^2^ = 0.563, df = 2, *p* = 0.002), *Cladosporium* (χ^2^ = 1.875, df = 2, *p* = 0.011), *Alternaria* (χ^2^ = 1.068, df = 2, *p* = 0.013), *Debaryomyces* (χ^2^ = 5.100, df = 2, *p* = 0.022), family *Didymosphaeriaceae* (χ^2^ = 1.457, df = 2, *p* = 0.031), *Vishniacozyma* (χ^2^ = 4.503, df =2, *p* = 0.009), and other genera (χ^2^ = 2.656, df = 2, *p* = 0.043). On the other hand, *Candida* (Wilcoxon rank-sum test, W = 5, *p* = 0.013, r = −0.003, CI 95% −0.172 to 0.006) exhibited significantly higher abundance in frequently cleaned households.

## 4. Discussion

This research represents a novel study in Croatia, investigating the relatively underexplored subject of indoor microbiomes. It places a special emphasis on the dust mycobiome, an area that has received even less attention, providing new insights into fungal communities and their potential impacts on health and indoor environments. While microbiome studies have gained significant attention globally, the focus has mainly been on human and environmental microbiomes. Consequently, indoor microbiomes, particularly in the context of health conditions such as asthma, remain relatively understudied. This gap is especially evident in Croatia, where this research marks the first comprehensive study of the dust microbiome in indoor environments and its potential link to childhood health.

The genera identified in this study reveal diverse relationships between outdoor and indoor environments. The source of most fungal genera found in indoor dust predominantly traces back to outdoor environments, where fungi inhabit diverse ecosystems such as soil, air, water, plants, and decaying organic matter [10,19,38]. This connection between outdoor and indoor mycobiomes is reflected in previous studies, which have identified commonly reported taxa such as *Aspergillus*, *Penicillium*, *Cladosporium*, *Alternaria*, *Epicoccum*, and *Fusarium* as frequent components of house dust worldwide. Additionally, yeast genera such as *Aureobasidium*, *Saccharomyces*, *Candida*, *Cryptococcus*, and *Rhodotorula* have been noted, alongside *Wallemia*, *Debaryomyces*, and *Malassezia*, which are often detected in indoor environments, including homes, schools, and office buildings [7,9,11,12,16,17,19,39,40,41]. Our findings align with these observations, as the top 10 genera identified in our study are mostly associated with outdoor environments (*Cladosporium*, *Alternaria*, *Debaryomyces*, *Saccharomyces*, *Cyberlindnera*, *Aureobasidium*, *Aspergillus*, and families *Didymosphaeriaceae* and *Didymellaceae*) [8,21,42,43,44,45,46,47,48,49,50]. However, two genera, *Saccharomyces* and *Debaryomyces*, are linked to human-related activities as well [8,51,52]. Several of these genera contain allergenic species, including *Alternaria*, *Cladosporium*, *Aspergillus*, and *Malassezia*, which are known for their potential to trigger respiratory symptoms [17].

These fungi are introduced into indoor spaces through various means, including air exchange, human activity, and pet interactions. One of the most notable genera in our findings is *Malassezia*, a human-associated fungus. *Malassezia* species are known for their reliance on lipids for growth, which restricts them to sebaceous areas of the human body, such as the scalp, face, and upper body [53]. The significant abundance of this genus under varying environmental conditions, as depicted in relative abundance plots, shows the significant contribution of human occupants to indoor fungal communities. Previous studies have similarly identified *Malassezia* as a major component of indoor dust microbiota, noting its relevance to studies on human–environment interactions [8,54]. Interestingly, our findings reveal that *Malassezia* has a higher relative abundance in households without pets compared to those with pets, likely due to its strong association with human skin. In pet-owning households, the introduction of other fungal taxa by pets may reduce the dominance of *Malassezia* through competition or ecological interactions. Similar trends have been reported in the study, where an increased number of pets correlated with a higher relative abundance of outdoor-associated genera and a decline in human-associated genera [41]. Beyond its environmental presence, *Malassezia* yeasts have been significantly associated with asthma and, more recently, with exacerbations in cystic fibrosis [6,25,55].

The two taxa with the highest abundance, following *Malassezia*, are *Cladosporium* and members of the family *Didymellaceae*. *Cladosporium* is known aeroallergen linked to allergic diseases, including asthma [17,47,56]. *Cladosporium* is present in both indoor and outdoor environments, suggesting its ability to thrive under varying conditions. Its presence in indoor environments is often associated with outdoor air and climatic factors, particularly damp conditions, which can negatively affect indoor air quality [8,45]. Our associations show that *Cladosporium* is linked with seasonality. Namely, its abundance favors damp conditions, as it has been observed to be more prevalent in non-heating conditions (Figures S1 and S2). The family *Didymellaceae*, on the other hand, is distributed across diverse hosts and habitats, including plants and outdoor air, which may explain its presence in indoor dust [43].

Among the other dominant genera were *Alternaria*, *Debaryomyces*, *Saccharomyces*, *Cyberlindnera*, *Aspergillus*, and members of the *Didymosphaeriaceae* family. *Alternaria* and *Aspergillus* are well-known allergenic fungal genera with widespread environmental distribution [17,57,58]. *Alternaria* is an airborne cosmopolitan fungus commonly found in soil, decaying plant material, and carpet dust, particularly prevalent in rural areas [21,46]. *Aspergillus* is among the most common fungal genera globally, thriving in indoor damp environments, and is associated with asthma, rhinitis, and eczema [8,46,59]. The genus *Cyberlindnera* is broadly distributed and often associated with soil and decomposition processes [48]. Its presence in indoor spaces, particularly damp environments, aligns with findings from studies of water-damaged classrooms [9]. Members of the genera *Debaryomyces* and *Saccharomyces* are linked to human-related activities and environments. Both genera are commonly found in the human gut and on children’s skin [25,51]. Additionally, the genus *Saccharomyces* is globally distributed and has been detected in soil, seawater, and various foods [42], while *Debaryomyces* has been identified in clinical samples [8,52]. In our findings, *Saccharomyces* demonstrated a significantly higher relative abundance in urban green environments compared to rural and urban built environments (Figures S3 and S4). The observed trends may be possibly due to the influence of vegetation and soil contact. Such environments offer a rich diversity of substrates, which are favorable for the proliferation of *Saccharomyces* [42]. In addition, *Saccharomyces* was linked to heating conditions, with a higher abundance during the heating season. This could be due to the indoor microclimatic changes caused by heating, such as reduced humidity and increased temperatures, which may favor its proliferation. As for *Debaryomyces*, the genus shows higher abundance in households with both frequent and rare/never dusting, while lower levels are observed with weekly dusting. This trend may result from dust dynamics, such as rare/never dusting, which allows for fungal spores to accumulate, while frequent dusting redistributes spores, maintaining their presence. In contrast, weekly dusting may disrupt this balance, reducing its abundance. Since *Debaryomyces* is commonly found in dust and is associated with skin [8,25,39,51], a substantial component of indoor dust, its prevalence in these environments is to be expected.

Pets and siblings appear to have the most significant influence on fungal diversity and community composition, as suggested by previous research [7,11,12,13,16,17,18,40,41,44]. Pets, known to transport spores and microorganisms from outdoor environments into indoor spaces via their fur and activities, are associated with increased fungal diversity. Similarly, siblings contribute to microbial exchange through their interactions, further shaping the indoor fungal landscape [17,18] with a higher number of siblings corresponding to increased abundance of certain fungal genera (Figure S1). This prompted our investigation into their combined effect on fungal taxa; *Aureobasidium* and *Vishniacozyma* were significantly more abundant in pet-owning households with one sibling, indicating the combined contributions of pets and sibling activity in introducing and sustaining these taxa. *Aureobasidium*, a genus commonly associated with outdoor environments [54,60], has also been identified on pet fur. Our association can be explained using this relationship: pets act as vectors for transporting outdoor-associated fungi into indoor spaces [61]. *Vishniacozyma* has been detected in bedroom dust samples before, and is associated with the presence of dogs and dog allergens, showing its potential link to pet-related dynamics in indoor environments [60]. *Cyberlindera* demonstrated higher abundance in pet-owning homes with multiple siblings. As an outdoor fungus associated with the decomposition of plant material in nature [48], and one of the most abundant genera found in floor dust from 50 elementary schools [9]; this result is consistent with previous research.

Cleaning practices emerged as another critical factor influencing fungal abundance and community structure. Specific activities such as vacuuming mattresses showed interesting variations in composition of genera (Figure S5). Frequent cleaning can selectively modify microbial profiles, often reducing diversity [24], thus potentially limiting beneficial microbial exposures. The combined influence of dusting and vacuuming practices on fungal communities demonstrated significant influence on at least nine taxa. The genus *Candida* was more abundant in households within the weekly dusting and frequent vacuuming category. This genus, along with *Debaryomyces* and *Malassezia*, commonly associated with the anthropogenic influences, indicate the connection between human activity and fungal presence in indoor environments. Additionally, *Cladosporium*, *Alternaria*, and members of the family *Didymosphaeriaceae* exhibited higher abundance in households with infrequent cleaning, suggesting that less frequent cleaning practices may create conditions favorable for fungal growth, such as the accumulation of organic material and sustained moisture levels [21]. However, the contrasting results observed among cleaning habit groups may be attributed to type of vacuum cleaners. Vacuum cleaners vary significantly in their ability to disperse airborne particles, including fungal spores, and this is an important factor to consider when assessing their impact on indoor air quality, as noted by Beguin and Nolard [21]. However, the extent to which frequency of cleaning practices shapes fungal communities remains relatively unexplored. This gap in knowledge shows the significance of this study, as it provides novel insights into the impact of cleaning habits on the indoor fungal microbiome.

Although effect sizes were calculated and presented to estimate the practical significance of the results, they should be interpreted with caution. Due to the complexity of microbial ecology and variability in fungal abundances, statistical effect sizes may not necessarily imply biological significance.

Socioeconomic variables also shaped fungal community composition (Figures S6 and S7). Lower-income households exhibited the highest alpha diversity, while higher-income households had the lowest. This suggests that lower-income environments may harbor more diverse fungal communities, potentially due to poorer ventilation, increased humidity, or greater exposure to outdoor contaminants [14,62]. Lower-income households also exhibited higher levels of fungal allergens, such as genera *Malassezia* and *Debaryomyces*, often associated with poor ventilation and damp conditions. These findings highlight the sensitivity of fungal communities to socioeconomic disparities and align with previous research on fungal exposure and environmental conditions [15].

When comparing indoor fungal microbiomes in Croatia to other countries, certain patterns appear, such as the dominance of *Cladosporium* and *Malassezia* in indoor environments. However, regional differences in climate, building materials, ventilation, and household behaviors likely contribute to different microbial compositions. While many outdoor-associated genera observed in this study are also common in other European and North American homes, the impact of socioeconomic disparities on fungal diversity may vary across countries due to differences in housing conditions, climate, and cultural cleaning practices.

## 5. Conclusions

This study presents a pioneering analysis of the mycobiome within the Croatian dust microbiome, offering valuable insights into how environmental, household, and socioeconomic factors shape fungal community composition in indoor environments. The genera *Malassezia*, *Cladosporium*, and family *Didymosphaeriaceae* emerged as the dominant taxa, with *Malassezia* particularly associated with human occupancy and resilience to cleaning practices.

We found that pet ownership, number of siblings, and cleaning habits are key drivers of fungal diversity and community structure, with pet households exhibiting higher fungal diversity and outdoor-associated genera. This study also emphasizes the role of socioeconomic factors in shaping fungal communities; households with higher socioeconomic status (income and education) are associated with increased diversity, while lower-income households showed higher levels of potentially allergenic fungi, reflecting disparities in living conditions. Cleaning practices also significantly impacted fungal diversity, with frequent cleaning reducing diversity, but failing to eliminate resilient genera such as *Malassezia*.

This study underscores the importance of considering indoor fungal diversity in understanding the potential health implications of microbial exposure, as well as the significant role of the environmental and behavioral factors that impact the household mycobiome.

## Figures and Tables

**Table 1:** Alpha diversity in relation to demographic, socioeconomic, and environmental factors.

Category	Variable	Total (n, %)	Alpha Diversity (*p*-Value)
Education parents	Basic	23 (25.6)	0.262
	Graduate	48 (53.3)	
	Master/PhD	19 (21.1)	
Household monthly income	<EUR 1500	7 (7.8)	
	EUR 1500–2500	25 (27.8)	0.018
	>EUR 2500	58 (64.4)	
	Suburban	19 (21.1)	0.548
Living environment	Urban (built surroundings)	35 (38.9)	
	Urban (green spaces)	36 (40.0)	
Type of home	House	30 (33.3)	0.451
	Apartment	60 (66.7)	
	None	12 (13.3)	
Number of siblings	1	54 (60.0)	0.239
	2 or more	24 (26.7)	
Pet ownership	No	61 (67.8)	0.117
	Yes	29 (32.2)	
	Occasionally	15 (16.7)	0.543
Dusting furniture	Once a week	45 (50.0)	
	Frequently	30 (33.3)	
	Never or once a year	19 (21.1)	0.193
Mattress vacuuming	Occasional	43 (47.8)	
	Frequent	28 (31.1)	
Season of sample collection	Heating	52 (57.8)	0.218
	No heating	38 (42.2)	

## Data Availability

The authors confirm that additional data supporting the findings of this study will be shared upon reasonable request.

## Supplementary Materials

The following supporting information can be downloaded at: https://www.mdpi.com/article/10.3390/jof11040261/s1, Figure S1. Relative abundance of microbial genera by household characteristics. (a) Stacked bar plot highlights the top 10 genera, ordered by mean abundance across samples for Heating season. (b) Violin plot illustrates genus-level distribution and variability, with *p*-values indicating significant differences between groups for Heating season. (c) Stacked bar plot highlights the top 10 genera, ordered by mean abundance across samples for Number of siblings. (d) Violin plot illustrates genus-level distribution and variability, with *p*-values indicating significant differences between groups for Number of siblings. Figure S2. Fungal genera relative abundance interaction with Household characteristics. (a) Interaction for Pets in the household and Heating season. (b) Interaction for Number of siblings and Heating season. Figure S3. Relative abundance of microbial genera by living conditions. (a) Stacked bar plot highlights the top 10 genera, ordered by mean abundance across samples for Living environment. (b) Violin plot illustrates genus-level distribution and variability, with *p*-values indicating significant differences between groups for Living environment. (c) Stacked bar plot highlights the top 10 genera, ordered by mean abundance across samples for Home type. (d) Violin plot illustrates genus-level distribution and variability, with *p*-values indicating significant differences between groups for Home type. Figure S4. Fungal genera relative abundance interaction with Living conditions. Figure S5. Relative abundance of microbial genera by household characteristics. (a) Stacked bar plot highlights the top 10 genera, ordered by mean abundance across samples for Mattress vacuuming frequency. (b) Violin plot illustrates genus-level distribution and variability, with *p*-values indicating significant differences between groups for Mattress vacuuming frequency. Figure S6. Relative abundance of microbial genera by socioeconomic factors. (a) Stacked bar plot highlights the top 10 genera, ordered by mean abundance across samples for Parental education. (b) Violin plot illustrates genus-level distribution and variability, with *p*-values indicating significant differences between groups for Parental education. (c) Stacked bar plot highlights the top 10 genera, ordered by mean abundance across samples for Household monthly income. (d) Violin plot illustrates genus-level distribution and variability, with *p*-values indicating significant differences between groups for Household monthly income. Figure S7. Fungal genera relative abundance interaction with socioeconomic characteristics.
