# Supplementary material for: Associations Between Indoor Fungal Community Structures and Environmental Factors: Insights from the Evidence-Driven Indoor Air-Quality Improvement Study
Source: J Fungi (Basel). 2025 Mar 28;11(4):261. doi: 10.3390/jof11040261 (PMC12028660; doi:10.3390/jof11040261)
Supplement: Supplementary file 1 [file jof-11-00261-s001.zip › jof-3491965-supplementary.pdf]

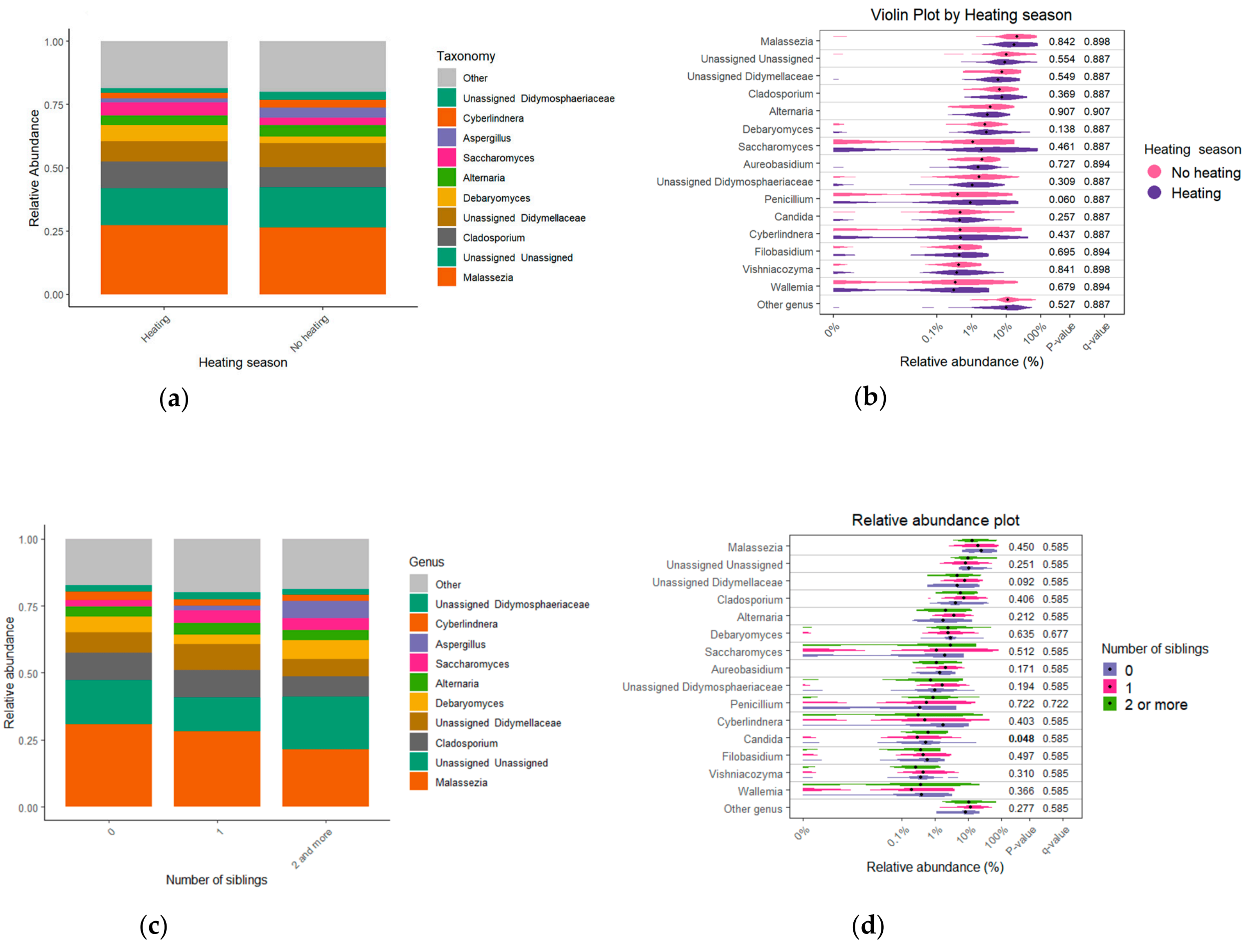

Figure S 1. Relative abundance of microbial genera by household characteristics. (a) Stacked bar plot highlights the top 10 genera, ordered by mean abundance across samples for Heating season. (b) Violin plot illustrates genus-level distribution and variability, with p-values indicating significant differences between groups for Heating season. (c) Stacked bar plot highlights the top 10 genera, ordered by mean abundance across samples for Number of siblings. (d) Violin plot illus-trates genus-level distribution and variability, with p-values indicating significant differences between groups for Number of siblings.

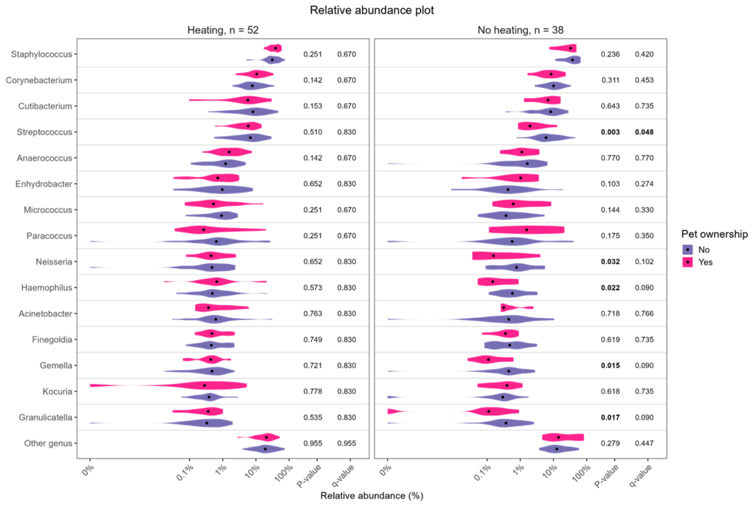

(**a**)

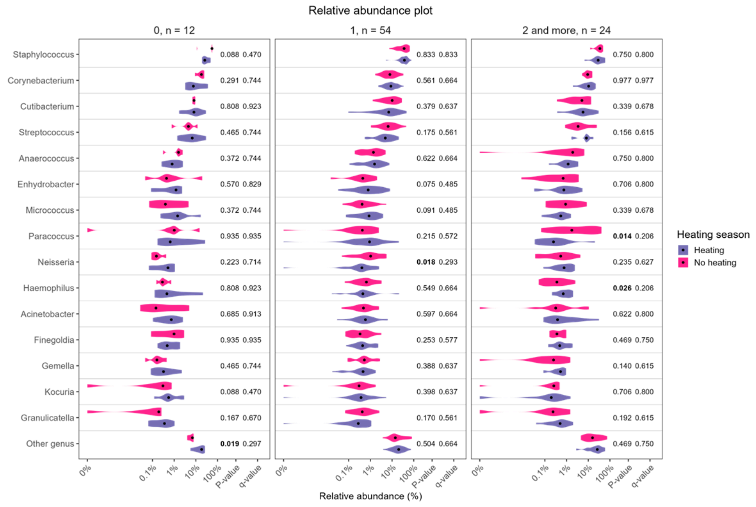

(b)

Figure 2. Fungal genera relative abundance interaction with Household characteristics. (a) Interaction for Pets in the household and Heating season. (b) Interaction for Number of siblings and Heating season.

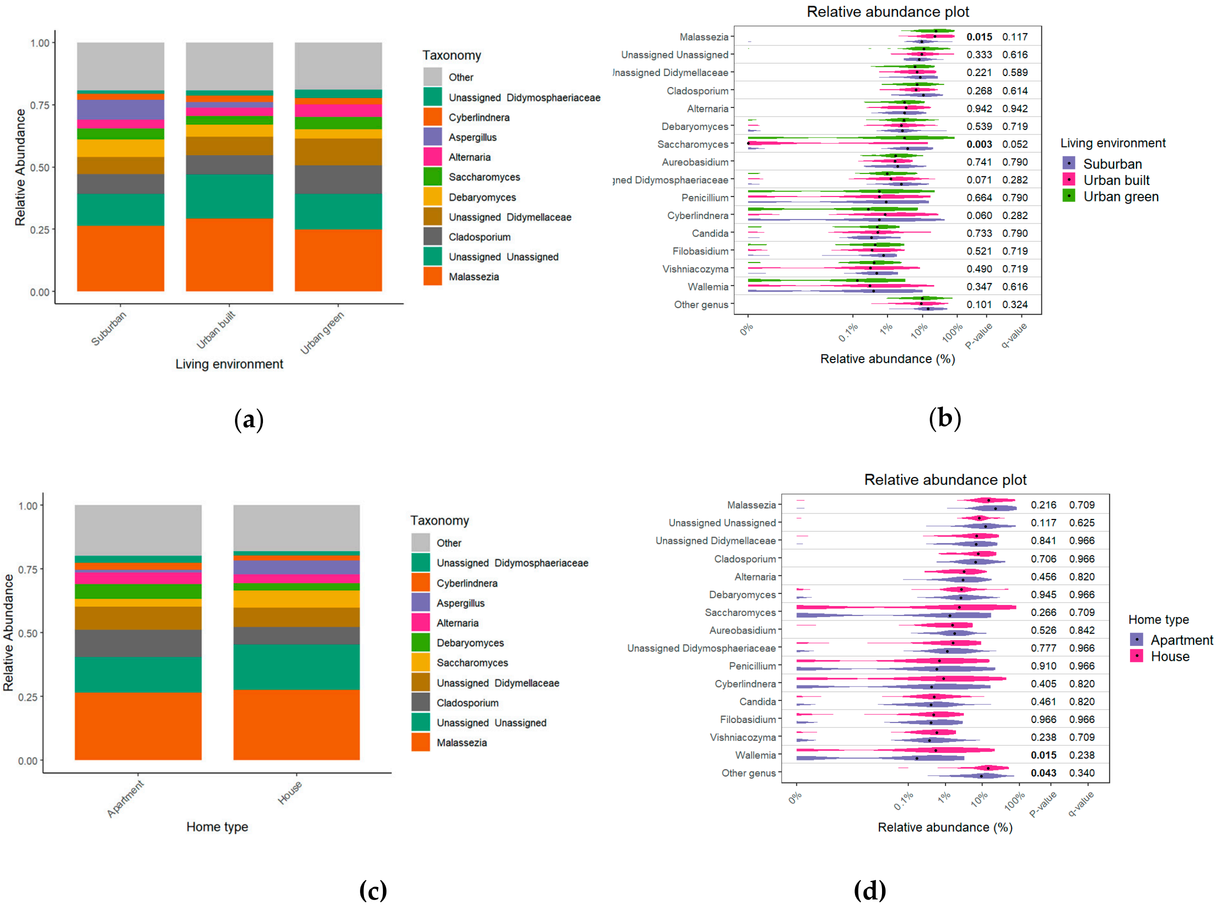

Figure S 3. Relative abundance of microbial genera by living conditions. (a) Stacked bar plot highlights the top 10 genera, ordered by mean abundance across samples for Living environment. (b) Violin plot illustrates genus-level distribution and variability, with p-values indicating significant differences between groups for Living environment. (c) Stacked bar plot highlights the top 10 genera, ordered by mean abundance across samples for Home type. (d) Violin plot illustrates genus-level distribution and variability, with p-values indicating significant differences between groups for Home type.

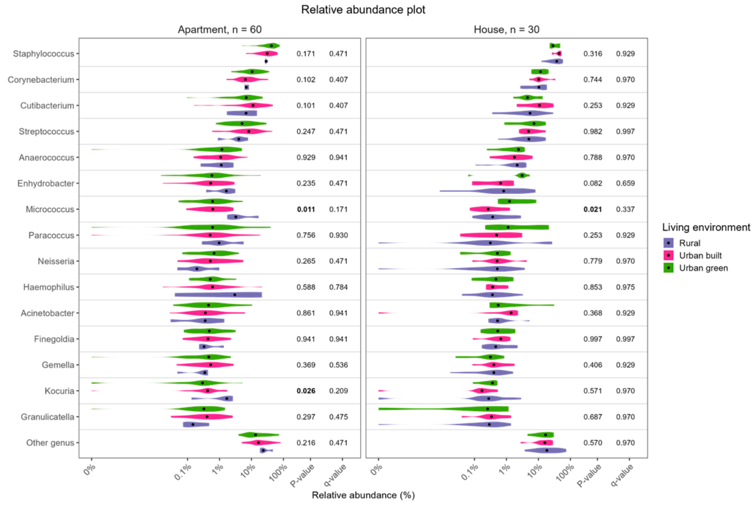

Figure S 4. Fungal genera relative abundance interaction with Living conditions.

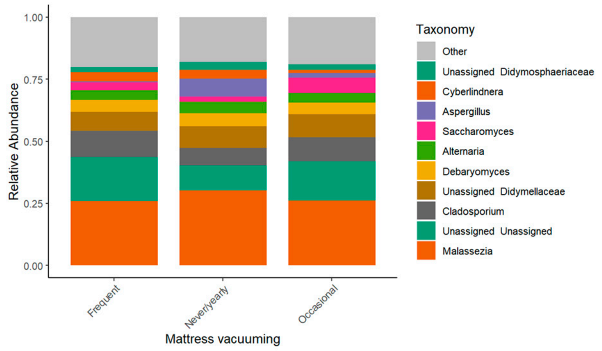

(a)

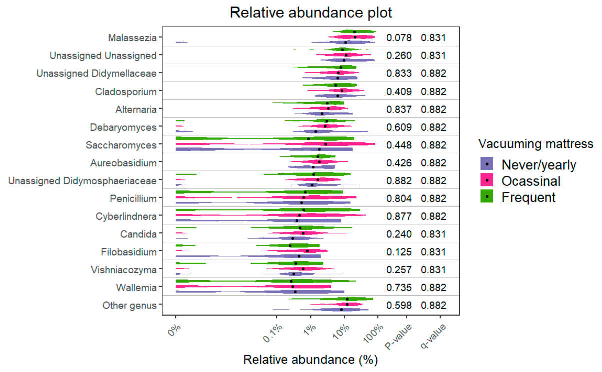

(b)

Figure S 5. Relative abundance of microbial genera by household characteristics. (a) Stacked bar plot highlights the top 10 genera, ordered by mean abundance across samples for Mattress vacuuming frequency. (b) Violin plot illustrates genus-level distribution and variability, with p-values indicating significant differences between groups for Mattress vacuuming frequency.

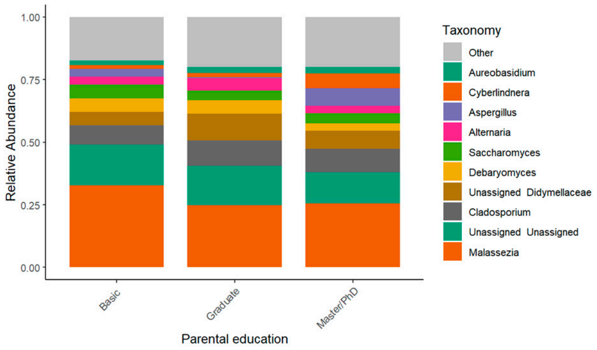

(a)

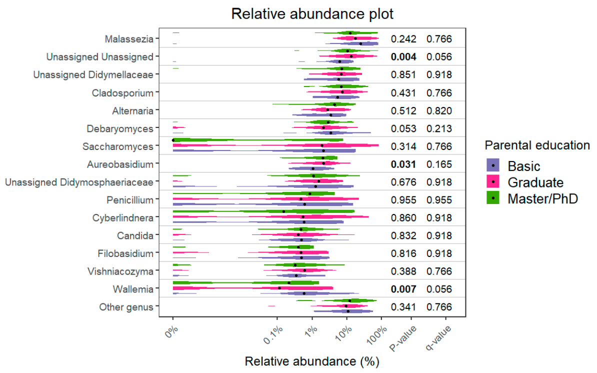

(b)

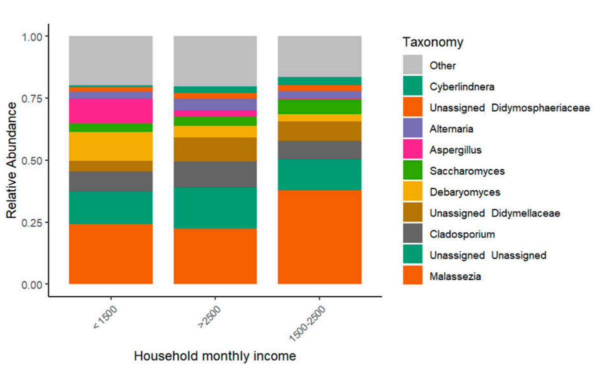

(c)

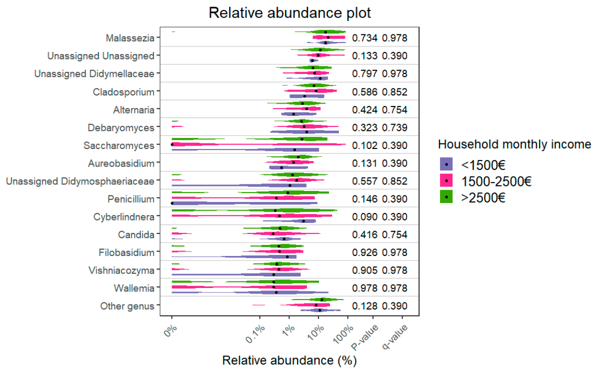

(d)

Figure S 6. Relative abundance of microbial genera by socioeconomic factors. (a) Stacked bar plot highlights the top 10 genera, ordered by mean abundance across samples for Parental education. (b) Violin plot illustrates genus-level distribution and variability, with p-values indicating significant differences between groups for Parental education. (c) Stacked bar plot highlights the top 10 genera, ordered by mean abundance across samples for Household monthly income. (d) Violin plot illustrates genus-level distribution and variability, with p-values indicating significant differences between groups for Household monthly income.

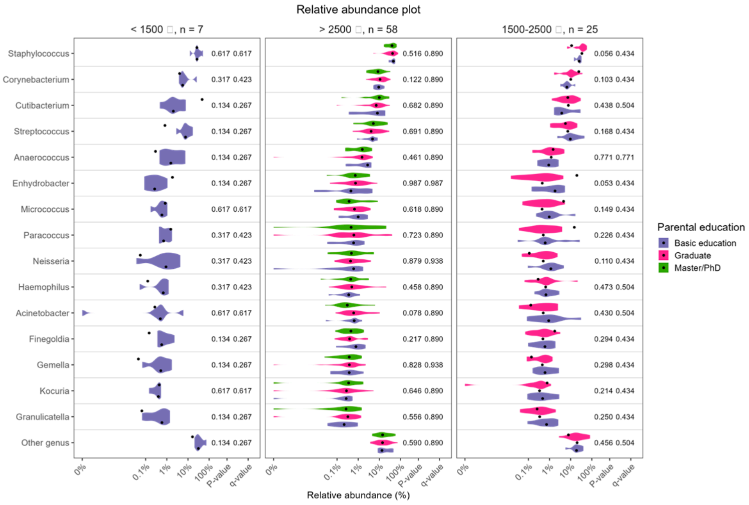

Figure S 7. Fungal genera relative abundance interaction with socioeconomic characteristics.
